# Exercise Preconditioning Ameliorates Cognitive Impairment in Mice with Ischemic Stroke by Alleviating Inflammation and Modulating Gut Microbiota

**DOI:** 10.1155/2022/2124230

**Published:** 2022-10-10

**Authors:** Heng Lv, Shasha Wang, Meihui Tian, Liya Wang, Jie Gao, Qitao Zhao, Zhaoyu Li, Xianjie Jia, Ying Yu

**Affiliations:** ^1^Department of Epidemiology and Statistics, School of Public Health, Bengbu Medical College, Bengbu 233000, China; ^2^Key Laboratory of Cardiovascular and Cerebrovascular Diseases, Bengbu Medical College, Bengbu 233000, China; ^3^Department of Physiology, School of Basic Medicine, Bengbu Medical College, Bengbu 233000, China

## Abstract

Several studies have demonstrated that exercise preconditioning is an effective means of alleviating poststroke cognitive impairment (PSCI). Mechanisms of regulating cognitive function have not been fully elucidated. Herein, the present study is aimed at exploring the effect of the microbiota-gut-inflammasome-brain axis in the process of exercise preconditioning moderating cognitive impairment after ischemic stroke. We observed that exercise preconditioning decreased infarct size, reduced the degree of neuronal damage, and alleviated cognitive impairment in mice with ischemic stroke. In addition, exercise preconditioning also reduced the expression of inflammatory cytokines, as well as NLRP3, Caspase-1, IL-18, and IL-1*β* protein expressions. Ischemic stroke could downregulate the abundance of *Roseburia* while increasing the abundance of the *Helicobacter* at the level of genus. As a comparison, exercise preconditioning increased the abundance of the *Lactobacillus*, which was beneficial for mice at the genus level. In conclusion, exercise preconditioning can improve cognitive dysfunction after ischemic stroke through alleviating inflammation and regulating the composition and diversity of the gut microbiota, which might provide a new strategy for the prevention of PSCI.

## 1. Introduction

Ischemic stroke is an emergency situation caused by reduced blood flow to the brain, which results in impairment to brain cells [1]. Poststroke cognitive impairment (PSCI) is one of the common consequences. It also is a major contributor of long-term disability and reduced quality of life. In a community-based study in China, the prevalence of PSCI was shown to be 80.97% [2]. The pathogenesis of PSCI is complicated, and there is still no effective clinical treatment [3]. Exercise preconditioning as an effective strategy has been shown experimentally to be neuroprotective in stroke survivors [4]. However, the biological mechanisms and pathways through which exercise preconditioning promotes cognitive function have not been completely clarified.

Extensive research suggested that exercise may exert a neuroprotective effect by reducing neuroinflammation [5]. Studies from both humans and animals had demonstrated that appropriate exercise delays cognitive aging and neurodegeneration [6, 7]. A study [8] published in Nature showed that “runner plasma,” which was collected from voluntarily running mice and injected into sedentary mice, reduced baseline neuroinflammatory gene expression and experimentally induced brain inflammation. This finding confirmed the presence of anti-inflammatory exercise factors that are metastable, target the cerebral vasculature, and are beneficial to the brain. What is more, a population-based study observed that regular exercise preconditioning was associated with fewer ischemic stroke complications and better long-term function outcomes [9].

There is substantial evidence that the pathogenesis of PSCI is also associated with inflammatory response [10]. The inflammasome is an important multiprotein complex that functions during inflammatory immune responses. Components of the inflammasome contain the NOD-like receptor pyrin domain-containing 3 (NLRP3), which is a multiprotein signaling complex containing the NLRP3 scaffold, the adaptor protein PYCARD/ASC, and Caspase-1. The interaction between these proteins can flexibly regulate the constitutive function of inflammasome, ensuring that inflammasome is activated at appropriate occasions [11]. Overactivation of inflammation is known to play a pivotal role throughout cerebral ischemia, from early injury to postischemic tissue recovery [12]. Specifically, NLRP3 is regarded as one of the predominant inflammasomes and plentifully expresses in the brain. It plays a key role in recognizing cellular damage and modulating inflammatory responses to ischemic stroke [13]. A study [14] showed that cerebral ischemia-reperfusion in mice was followed by increased infarct area and hydrocephalus content and elevated NLRP3 and Caspase-1 expression. Systemic inflammation activates the NLRP3 inflammasome, triggers neuroinflammation, and exacerbates ischemic brain injury and cognitive impairment.

In recent years, there has been an increasing interest in the role of the microbiota-gut-brain (MGB) axis in modulating the brain function [15]. The MGB axis also plays a vital role in the pathophysiology of PSCI and managing inflammatory reaction [16]. It suggests that the gut microbiota exchanges information with the central nervous system through immune, neuroendocrine, and vagal “bidirectional brain-gut signals,” affecting the host's brain function and thus its behavior and cognitive function [17]. Furthermore, systemic low-grade chronic inflammation may be caused by dysbiosis of gut microbiota in stroke patients, which is a critical cause in the pathogenesis of PSCI [18]. Regulation of gut microbiota has been a latent target for treatment and prevention of some chronic diseases in the future. Numerous studies have indicated that the gut microbiota can be harmonized by a variety of factors such as exercise, antibiotics, infection, and diet [19–21]. Exercise can modulate the composition and diversity of gut microbiota [22]. Exercise-induced alterations in the gut microbiome are associated with corresponding physiological changes in the host, including immunity and metabolism [23]. Overgrowth of hazardous microbiota induces inflammation by altering the intestinal mucosal barrier, leading to neuroinflammation and neurodegeneration in the central nervous system [24]. Notably, recent evidence supports that the NLRP3 inflammasome has a key role to play in orchestrating host physiology and formatting the peripheral and central inflammatory/immune reactions to central neurological diseases through the release of IL-18 and IL-1*β* [25, 26]. The gut microbiota may interact with the NLRP3 inflammasome through a dynamic interaction, known as the microbiota-gut-inflammasome-brain axis [27]. Gut microbiota adopt inflammasome signal to regulate peripheral inflammatory pathways, which in turn helps to maintain brain homeostasis.

Although studies have showed that exercise has a positive effect on the gut microbiota, it is not known whether the microbiota-gut-inflammasome-brain axis plays a role in exercise preconditioning with ischemic stroke. Therefore, the aims of the present study are as follows: (1) to investigate the effects of exercise preconditioning on the cognitive outcome of ischemic stroke and (2) to determine whether exercise preconditioning can improve cognitive function after stroke by inhibiting inflammation and regulating gut microbiota.

## 2. Materials and Methods

### 2.1. Animals

The Animal Center of Anhui Medical University (Hefei, China) provided us with 40 male C57BL/6J mice (age: 6-8 weeks; weight: 22-24 g). Mice were kept in a 12 h cycle of light/darkness, and water and food were available for free. The animal protocols were authorized by the Laboratory Animal Ethics Committee of Bengbu Medical College and were conducted in keeping with the ethical standards.

### 2.2. Experiment Protocol

C57BL/6J mice were evenly randomized into the sham operation group (Sham), the middle cerebral artery occlusion group (MCAO), the sham operation with exercise preconditioning group (EP+Sham), and the MCAO with exercise preconditioning group (EP+MCAO) (*n* = 12 in each group). Mice in the EP+Sham group and the EP+MCAO group were kept in cages equipped with running wheels for rodents and made to exercise autonomously for 4 weeks. Mice in the Sham group and the MCAO group were maintained in conventional cages. Subsequently, the MCAO group and EP+MCAO were subjected to right brain ischemia-reperfusion operation, while the Sham group and EP+Sham group only exposed the right external carotid artery and common carotid artery via sham operation, without ischemia stroke in the right brain. After 24 h of reperfusion, the mice were stimulated to defecate by lifting their tails at hourly intervals between 9 a.m. and 12 a.m., and approximately 200 mg of feces was collected from each mouse [28]. Considering the effect of circadian rhythms on intestinal flora, all samples were collected in the same time period of days [29]. We stored the fecal pellets at -80°C until further processing.

### 2.3. Middle Cerebral Artery Occlusion (MCAO)

It was performed to induce an ischemic stroke in mice by occluding the middle cerebral artery. Mice were given anesthesia by intraperitoneal injection of sodium pentobarbital (100 mg/kg). The right common carotid artery, internal carotid artery, and external carotid artery were separated through the midline neck incision. A 30 mm long and 0.12 mm thick nylon monofilament (MSMC21B120PK50, RWD Life Science, Shenzhen, China), with its tip rounded by silica gel (4 mm in length and 0.21 mm in diameter), was inserted and left for 90 min in the internal carotid artery from the common carotid artery to the beginning of the middle cerebral artery. After 90 min, the monofilament was retracted to restore reperfusion after cerebral ischemia. Mice of the Sham group and EP+Sham group received the same procedure, except for monofilament penetration.

### 2.4. Neurologic Functional Scoring

The neurological function of mice was examined after 24 h of reperfusion with a five-level grading system according to Longa: 0, no deficits; 1, inability to extend the right paw; 2, longitudinal rotation; 3, falling to the right; and 4, inability to walk spontaneously. Mice that scored between 1 and 3 neurologically were selected for the following study, while mice that scored equal to 1 or 4 were considered unsuccessful for MCAO surgery. Our inclusion criteria were as follows: (1) a neurological score from 1 to 3 based on Longa's grading system and (2) at least 5% loss in body weight at 24 h after ischemic stroke [30]. The animals that failed to satisfy one of these criteria at 24 h poststroke were deemed spontaneously recovered and excluded.

### 2.5. Behavior Testing

#### 2.5.1. Novel Object Recognition Task

An assessment of mice's nonspatial recognition memory capacity can be made through the novel object recognition (NOR) task. In this task, the day before the exact test execution, mice were habituated to the test space for 30 min to reduce stress responses [31]. In the training phrase, mice were positioned in an opening field with two identical objects. For a period of 10 min, mice were asked to explore the same objects at the same distance on a familiar arena. After 1 h, the mice were set back in the same arena in front of two objects, one of which was swapped with a new object, for another 5 min. The time spent by the mice adventuring the two objects was marked. And the odor of particular mouse was removed with spraying ethanol before testing the next one. *N* (novel) was the number of times mice probe for new objects, *F* (familiar) was the number of times they explored familiar objects, and discrimination index was calculated as *N*/(*N* + *F*).

#### 2.5.2. Y-Maze Test (Spontaneous Alternation)

The spontaneous alternation experiment is used as a method to detect spatial recognition memory capacity in rodents by exploiting their curiosity for novelty. They prefer to explore areas that they have never been to before. The test was performed in a symmetrical white Y-maze with three arms (length 20 cm × width 10 cm × height 20 cm). The mice were posed at the very end of one arm of a Y-shaped maze and permitted to move freely for a period of 8 min. A series of arm entries were visually evaluated and scored by experimenters who were blinded to the treatment. One alternation was determined as entering all three arms consecutively. The maximum number of alternations was equal to the total number of arms entered minus 2. The spontaneous alternation rate was calculated as actual number of alternations/maximum number of alternations.

### 2.6. Brain Infarct Volume

After scoring neurological function, mice were profoundly anesthetized with pentobarbital sodium (600 mg/kg) to isolate the brain quickly, and the brains were placed in a -20°C refrigerator for 15 minutes. Subsequently, the brains from Bregma +4.0 mm to 6.0 mm were sliced into five 2.0 mm thick sections. Then, the sections were stained with 2,3,5-triphenyl tetrazolium chloride solution (TTC, Sigma-Aldrich, St. Louis, Missouri, USA) in a 37°C water bath for 30 minutes and then fixed with 4% formaldehyde for 15 minutes. The infarct area was identified by nonstaining region, while the live area should turn red. The infarct area was measured using ImageJ software. The relative infarct volume was manually calculated according to the following formula: infarct percentage = (volume of the contralateral hemisphere − volume of the noninfarct contralateral hemisphere)/volume of the contralateral hemisphere × 100%.

### 2.7. Morphological Examination

The mice were anesthetized and their brains were taken out after cardiac perfusion with phosphate-buffered saline (PBS) and 4% paraformaldehyde. The brains were fixed in 4% paraformaldehyde for overnight, embedded in paraffin, cut into 5 *μ*m thick sections, and stained with hematoxylin-eosin (HE). Histomorphology changes of the right hippocampus were observed under the microscope.

### 2.8. Enzyme-Linked Immunosorbent Assay (ELISA)

After 90 min of reperfusion, blood samples were gathered from venous plexus of fundus. The serum was separated by centrifugation at 3000 rpm for 15 min at 4°C and collected. The levels of IL-18 and IL-1*β* were examined using an IL-18 ELISA kit (Calvin Biotechnology, Suzhou, China) and an IL-1*β* ELISA kit (Calvin Biotechnology, Suzhou, China), following the instructions separately provided by the manufacturer. The absorbance of samples was measured at 450 nm.

### 2.9. Western Blot Analysis

The total proteins were extracted from the hippocampal region of mice, and the protein concentration was determined by BCA assay. Equivalent amounts of protein samples were sampled on a 10% SDS-PAGE gel for electrophoretic separation of the proteins, and then, the PVDF membranes were electrotransformed by constant current at 280 mA for 90 min. Subsequently, the membranes were soaked with 5% skim milk powder in TBST (Tris-buffered saline containing 1% Tween-20) for 2 h. Then, the corresponding primary antibody was added overnight at 4°C. The primary antibodies were NLRP3 (1: 1000, ab263899), Caspase-1(1: 1000, ab179515), IL-1*β* (1: 1000, ab234437), IL-18 (1: 1000, ab207323), and *β*-actin (1: 2000, BL005B) and were purchased from Abcam (Cambridge, UK) except *β*-actin which was purchased from Biosharp (Anhui, China). Then, membranes were removed, rinsed 3x for 10 minutes with TBST, and incubated for 2 h at 37°C with the goat anti-rabbit IgG (1 : 10000, BL003A, from Biosharp, Anhui, China), respectively. TBST was used to wash the membranes three times before they were subjected to Bio-Rad electrophoresis (Bio-Rad Laboratories, Hercules, CA, United States). Analyzing all band intensities was done using ImageJ software.

### 2.10. Gut Microbiota Analysis

The 16S rRNA method was used to detect gut microbiota as follows: genomic DNA was obtained from mouse feces using the manufacturer's designated DNA extraction kit (DNeasy PowerSoil Kit, Mo Bio, United States) and was quantified using Nanodrop. The quality of DNA extraction was confirmed by 1.2% agarose gel electrophoresis. PCR was performed using primer pairs (forward: ACTCCTACGGGAGGCAGCA; reverse: TCGGACTACHVGGGTWTCTAAT) against the highly mutated V3-V4 region of the bacterial 16S rRNA gene. PCR amplification was performed using Pfu high fidelity DNA polymerase (TransGen Biotech), and the number of amplification cycles was strictly controlled. Then, 25 *μ*l of PCR product was purified by adding 0.8x volume of magnetic beads (Vazyme VAHTSTM DNA Clean Beads). PCR amplification recovery products were subjected to fluorescence quantification using the Quant-iT PicoGreen dsDNA assay kit and a microplate reader for quantification (BioTek, FLx800). Sequencing libraries were prepared using the Illumina TruSeq Nano DNA LT Library Prep Kit. Double-ended sequencing of community DNA fragments was performed using the Illumina MiSeq platform. Chimeric sequences were screened using the DADA2 method. The Greengenes and Silva databases were selected for taxonomic annotation of species on the QIIME2 (2019.4) platform.

### 2.11. Statistical Analysis

The experimental results were presented as mean ± standard error (SEM). One-way ANOVA after Newman-Keuls test was used to analyze the data between multiple groups. *P* < 0.05 was deemed to indicate a statistically significant difference.

## 3. Results

### 3.1. Exercise Preconditioning Ameliorated Neurological Scores and Reduced Infarction Area in Mice with Ischemic Stroke

**Figure 1:**
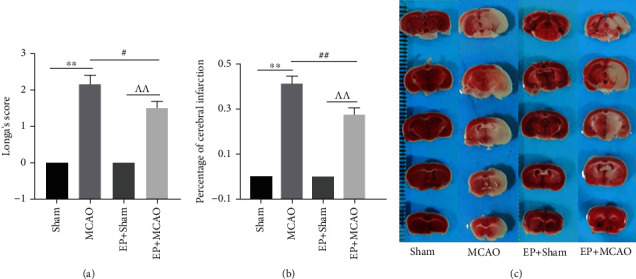
Effects of ischemic stroke and exercise preconditioning on neurological scores and infarction volume. (a) The neurological function scores of each group of mice were assessed at 24 h (*n* = 12). (b) Comparison of cerebral infarct volume of the ipsilateral brain between groups (*n* = 4). (c) Infarct size was determined by tetrazolium chloride (TTC) staining after cerebral ischemia-reperfusion (*n* = 4), and the infarct area was identified by nonstaining region, while the live area should turn red. ^∗∗^*P* < 0.01 vs. Sham; ^##^*P* < 0.01 vs. MCAO; ^^^^*P* < 0.01 vs. EP+Sham.

After successful induction of focal cerebral ischemia by the MCAO method, we assessed the effect of ischemic stroke and exercise preconditioning on neurological deficits by the Longa method. The results indicated no symptoms of neurological impairment in the Sham group and EP+Sham group. In contrast, the neurological deficit scores were significantly higher in the MCAO group mice than in the Sham group (*P* < 0.01, Figure 1(a)). Yet, four weeks of exercise preconditioning significantly reduced the score compared with the MCAO group (*P* < 0.05, Figure 1(a)). These results suggest that exercise preconditioning ameliorated the neurological damage which occurs after ischemic stroke in mice. Subsequently, we evaluated the infarct size. As shown in Figures 1(b) and 1(c), there were no infarction volume in the Sham group and EP+Sham group. The infarct area significantly appeared in the ischemia groups, and a statistically significant difference was found between the MCAO group and EP+MCAO group (*P* < 0.01, Figures 1(b) and 1(c)). Our data pointed to the fact that exercise preconditioning ameliorates neurological scores and the infarction area due to ischemic stroke.

### 3.2. Exercise Preconditioning Improved Cognitive Function in Mice with Ischemic Stroke

#### 3.2.1. Novel Object Recognition Task

**Figure 2:**
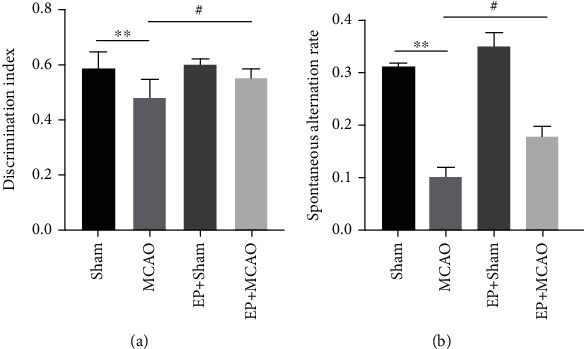
Effects of ischemic stroke and exercise preconditioning on the cognitive function of mice. (a) Discrimination index of mice in novel object recognition task (*n* = 7). (b) Spontaneous alternation rate of mice in Y-maze test of spontaneous alternation (*n* = 7). ^∗∗^*P* < 0.01 vs. Sham; ^#^*P* < 0.05 vs. MCAO.

A new object recognition task was utilized for evaluating nonspatial memory capacity, which is associated with the hippocampus. When compared to the Sham group, the mice in the MCAO group spend less time navigating new objects (*P* < 0.01, Figure 2(a)). In contrast, this reduced ability was enhanced by the exercise preconditioning (*P* < 0.05, Figure 2(a)). Ischemic stroke affects nonspatial recognition memory capacity in mice, while exercise preconditioning before ischemic stroke improves cognitive function.

#### 3.2.2. Y-Maze Test (Spontaneous Alternation Task)

Y-maze test of spontaneous alternation task was used to evaluate spatial memory capacity which is in connection with the hippocampus. The experimental results of Y-maze indicated that the mice in the MCAO model group had significantly reduced spontaneous alternations rate compared with the Sham group, and the difference was significant (*P* < 0.01, Figure 2(b)). While the mice given exercise preconditioning had increased rate of spontaneous alternations, it was remarkably higher compared to the MCAO model group (*P* < 0.05, Figure 2(b)), indicating that exercise preconditioning could improve the cognitive impairment caused by ischemic stroke and improve spatial memory ability.

### 3.3. Exercise Preconditioning Improves the Extent of Neuronal Damage in the Hippocampus of Mice with Ischemic Stroke

**Figure 3:**
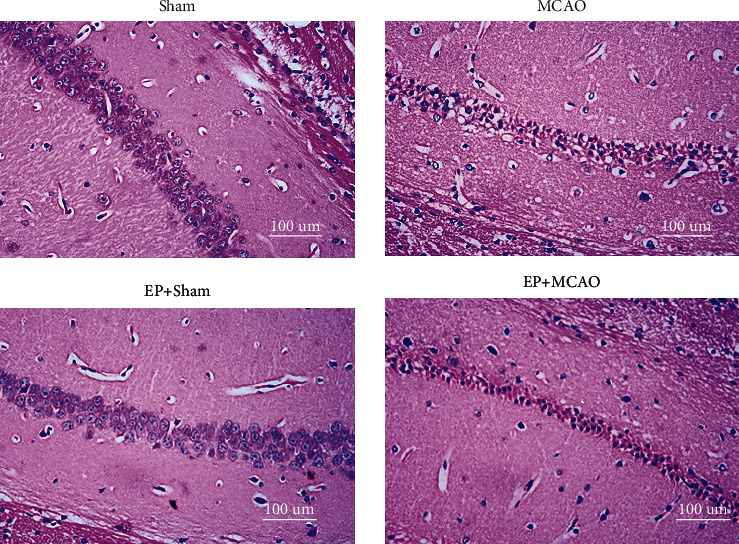
Effects of ischemic stroke and exercise preconditioning on the histopathological morphology of the hippocampal region (400x).

HE staining showed that hippocampal neurons of mice in the Sham groups were arranged neatly, with intact cell structure and visible nucleus. In contrast, MCAO mice exhibited significant neuronal damage with irregular cell shape, concentrated cytoplasm and nuclei, and damaged hippocampal structures. The damage was improved in the EP+MCAO group compared with the MCAO group, which indicated that exercise preconditioning could have a protective effect on brain tissue (Figure 3).

### 3.4. Exercise Preconditioning Mitigated the Expression of Inflammatory Factors Caused by Ischemic Stroke

**Figure 4:**
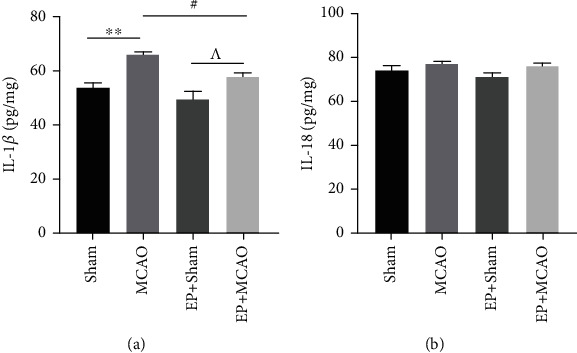
Effects of ischemic stroke and exercise preconditioning on proinflammatory cytokines in serum of each group. (a) The expression of IL-1*β* in brain tissue of mice (*n* = 8). (b) The expression of IL-18 in brain tissue of mice (*n* = 8). ^∗∗^*P* < 0.01 vs. Sham; ^#^*P* < 0.05 vs. MCAO; ^^^*P* < 0.05 vs. EP+Sham.

Since the inflammatory response is involved in the pathological process of ischemic stroke, we used ELISA to observe the alteration of IL-1*β* and IL-18 after ischemic stroke and if exercise preconditioning can modulate their excretion. The IL-1*β* level was elevated in the MCAO group when compared with the Sham group, as shown in Figure 4(a). In comparison with MCAO group, the significant decrease of IL-1*β* was presented by exercise preconditioning before surgery. Our data indicated that IL-1*β* is involved in ischemic stroke and that exercise preconditioning could reduce its expression. However, the levels of IL-18 in MCAO and EP+MCAO had an increasing trend compared with Sham groups, but not statistically significant (Figure 4(b)).

### 3.5. Exercise Preconditioning Reduced the Expression of NLRP3 Inflammasome and Proinflammatory Factors Induced by Ischemic Stroke

**Figure 5:**
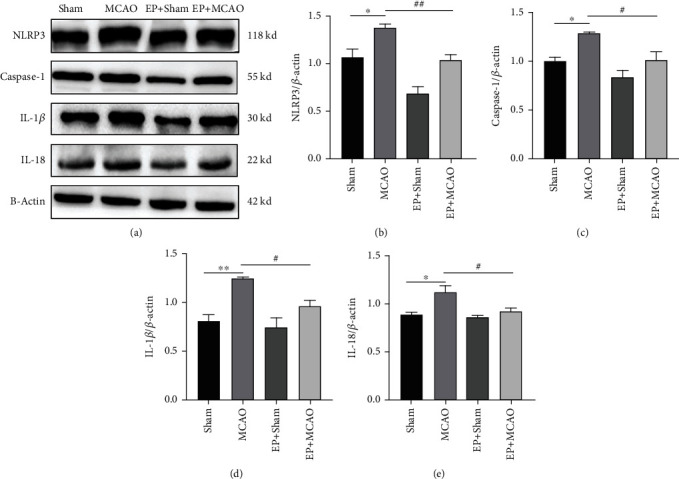
Representative western blots (a) and quantification data of NLRP3 (b), Caspase-1 (c), IL-1*β* (d), and IL-18 (e) for each group (*n* = 5). ^∗∗^*P* < 0.01 vs. Sham; ^#^*P* < 0.05 vs. MCAO.

To clarify the differences in expression of NLRP3 inflammasome and proinflammatory cytokines at the protein level among the groups, the protein expression levels of NLRP3, Caspase-1, IL-18, and IL-1*β* in ischemic brain tissue were determined (Figure 5). The protein expression levels of NLRP3, Caspase-1, IL-18, and IL-1*β* were significantly higher in the MCAO group compared with the Sham group. Exercise preconditioning reduced the expression levels of NLRP3, Caspase-1, IL-18, and IL-1*β* effectively. These findings suggested that exercise preconditioning attenuates inflammation in mice with ischemic stroke.

### 3.6. Exercise Preconditioning Regulated the Diversity and Composition of Gut Microbiota

**Figure 6:**
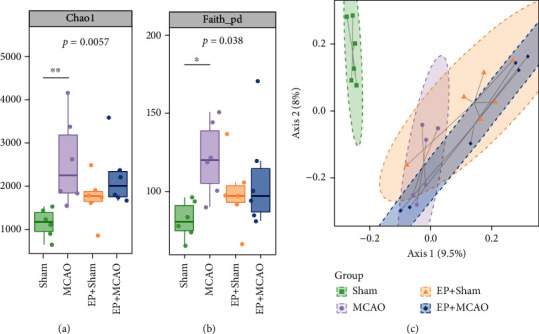
Ischemic stroke and exercise preconditioning affect the alpha and beta diversity of gut microbiota in mice (*n* = 6). (a) Chao1; (b) Faith's PD; (c) PCoA analysis.

We used 16S rRNA gene pyrosequencing to examine the differences in gut microbiota among all groups. The 1520869 clean sequences were generated through the high-throughput pyrosequencing. Firstly, we viewed the alpha diversity which includes Chao1 and Faith's PD indices that independently represent richness and evolution-based diversity. As illustrated in Figures 6(a) and 6(b), compared to the Sham group, the MCAO group had increased the levels of Chao1 and Faith's PD indices, indicating that ischemic stroke increased the richness and diversity of the species in mice. The differences in Chao1 and Faith's PD indices between the MCAO group and the EP+MCAO group were insignificant, indicating that exercise preconditioning did not significantly affect the richness and the alpha diversity of species.

The PCoA plot was subsequently used to analyze the beta diversity, where different samples exhibited clustering or scatter distributions, and samples with similar components were placed in proximity to each other in the plot. The results showed that (Figure 6(c)) the distribution of intestinal microbiota between the Sham and MCAO group as well as between the Sham and EP+Sham group was clearly separated, indicating that ischemic stroke can not only change the diversity of intestinal microbiota but also change the distribution structure of microbiota. Meanwhile, exercise preconditioning can also reshape the distribution of intestinal microbiota in mice.

**Figure 7:**
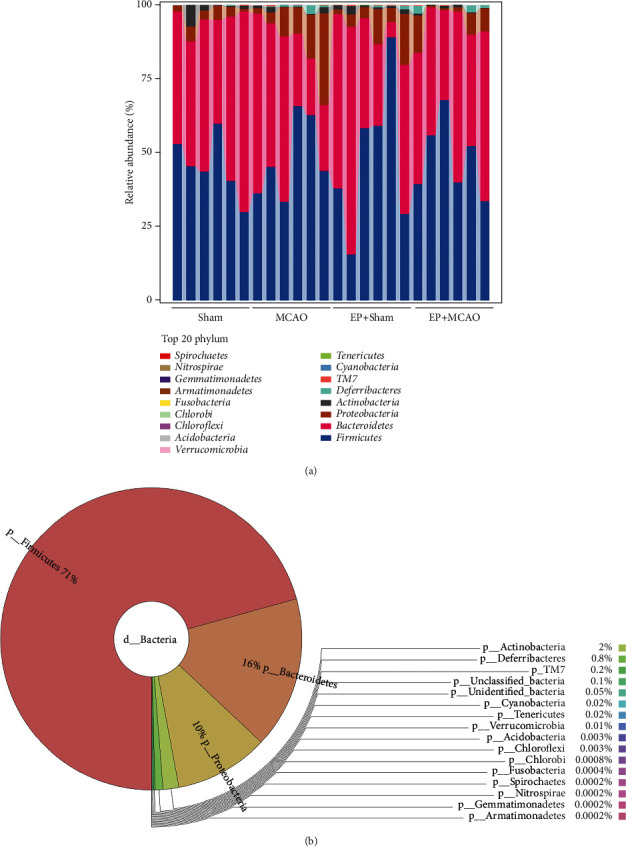
Phylum-level effects of ischemic stroke and exercise preconditioning on gut microbiota (*n* = 6). (a) The abundance of gut microbiota at the level of phylum. (b) Interactive presentation of sample taxonomic composition at the level of phylum.

Through clustering, we derived the religious abundance of each group of microbial communities at different taxonomic levels. Seventeen different phyla of gut microbiota were identified, with *Firmicutes* (71%), *Bacteroidetes* (16%), and *Proteobacteria* (10%) emerging as the most dominant phyla (Figure 7). About 97% of the overall bacterial abundance was constrained to these three phyla.

**Figure 8:**
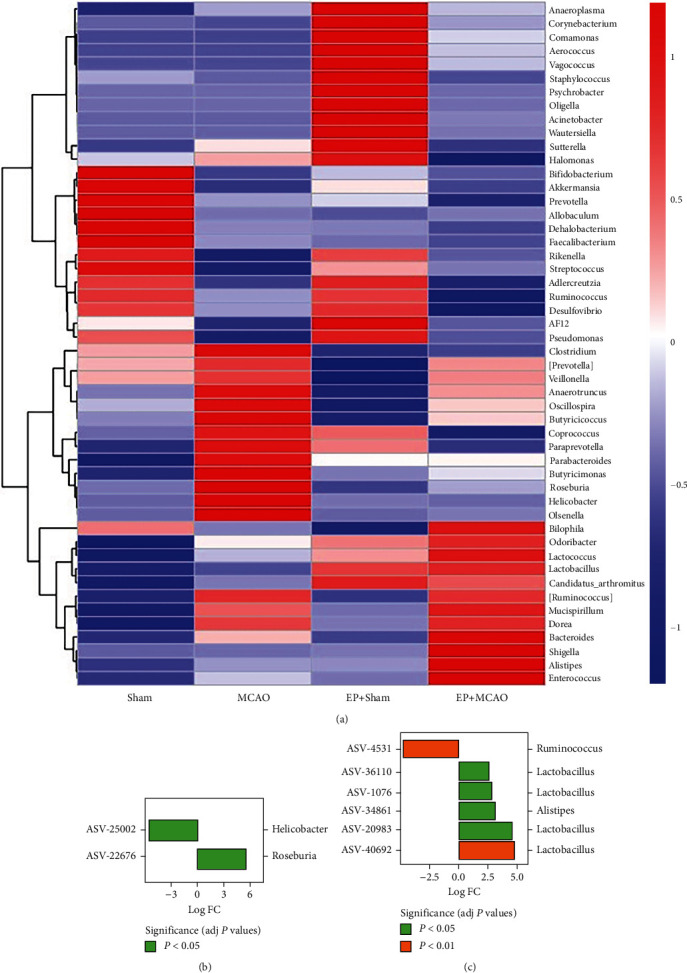
Effects of ischemic stroke and exercise preconditioning on the abundance of gut microbiota at the genus level (*n* = 6). (a) The abundance of gut microbiota at the genus level. (b) Log2 values of the multiple of ASV (fold change; FC) compared to the MCAO group in the Sham group, with positive values indicating upregulation, while negative values indicate downregulation. (c) Log2 values of the multiple of ASV (fold change; FC) in the EP+MCAO group compared to the MCAO group, with positive values indicating upregulation, while negative values indicate downregulation.

Furthermore, the gut microbiota composition at the genus level was analyzed.  Figure 8(a) tells the story. Hierarchical clustering analysis using the unweighted pair group method with arithmetic mean (UPGMA) showed that the majority of the samples were clustered within their own groups. Meanwhile, to find the ASV (amplicon sequence variants) with statistically significant differences between groups, we used the metagenomeSeq method to compare the samples two-by-two, called the fitFeatureModel function to fit the distribution of each ASV using a zero-inflated log-normal model, and used the fit results of this model to discriminate the significance of the differences. As shown in Figure 8(b), compared with the MCAO group, the *Helicobacter* in the Sham group was decreased and the *Roseburia* was increased. As shown in Figure 8(c), compared with the MCAO group, the *Ruminococcus* in the EP+MCAO group was decreased and the *Lactobacillus* and *Alistipes* were increased. Other ASV IDs not mentioned are not classified at the genus level.

**Figure 9:**
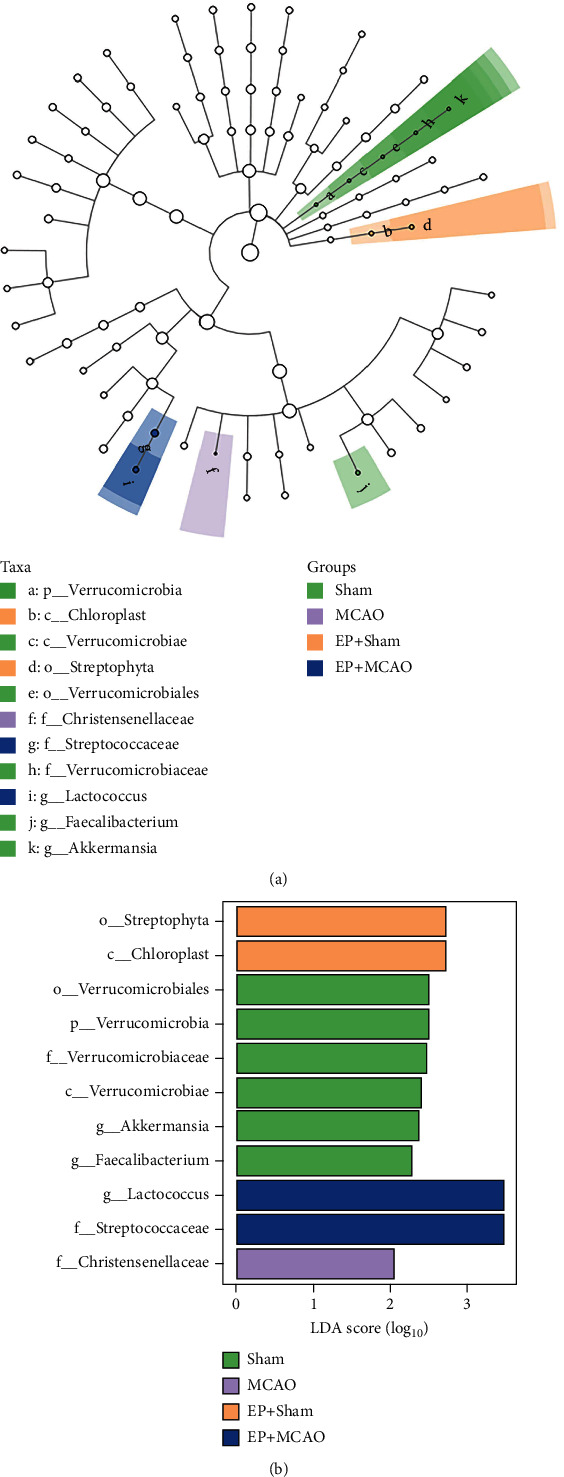
Linear discriminant analysis effect size. The biomarker of intergroup differences was found using LEfSe (*n* = 6). (a) The evolutionary groupings of different species are shown. Phylum to genus (or species) is depicted by a circle radiating from the center. Small circles represent different classification levels, and the diameter of the circle represents relative abundance for each level. (b) This graph illustrates the distribution of LDA values for species. In the bar chart, the colors denote groups, while the species contributions are shown by the length of the bars.

In the final analysis, LEfSe was queried for biomarkers of intergroup differences and species that differed significantly in the classification of the samples (Figure 9). Among the three differential biomarkers in terms of genus, *Akkermansia* and *Faecalibacterium* of the Sham group and Lactococcus of the EP+MCAO group were found.

## 4. Discussion

In the current study, we investigated the protective effect of exercise preconditioning against cognitive impairment in ischemic stroke. We provided direct evidence that exercise preconditioning decreased neurological deficits, infarct size in mice subjected to ischemic stroke. In addition, after exercise preconditioning, the expression of NLRP3 inflammasome was reduced, the composition and the beta diversity of gut microbiota were remodeled, and the impairment of cognitive function was alleviated in ischemic stroke. These findings indicated that exercise preconditioning improved cognitive dysfunction after ischemic stroke through alleviating inflammation and modulating the composition and diversity of gut microbiota. According to our study, we provided a new pathophysiological viewpoint on exercise preconditioning to cognitive impairment in ischemic stroke.

PSCI is one of the main complications after stroke, and the prognostic effect of treatment is restricted. Previous studies have shown that neuroinflammation is considered to be an important factor in PSCI [31]. Inflammasomes have been taken as therapeutic targets in human diseases [32]. The NLRP3 inflammasome is an intracellular multiprotein complexity that induces sets of proinflammatory chemokines, leading to inflammation. In the central nervous system, NLRP3 inflammasome was first reported to be activated in cortical neurons under ischemic conditions, and the expressions of NLRP3, Caspase-1, and IL-1*β* were upregulated in vitro and in vivo [33]. In our study, the levels of inflammatory factors including NLRP3, Caspase-1, and IL-1*β* were elevated in the MCAO group, demonstrating that the NLRP3 inflammasome was associated with the development of ischemic stroke. Nevertheless, the expression of IL-18 was not significantly different in the four groups. The reason is that the expression of IL-18 exhibits a delayed induction time process, starting from 24 to 48 h and peaking 6 days after ischemia [34]. Furthermore, we also observed that the cognitive function was impaired in the MCAO group of the mice, and the morphology and structure of neurons in the hippocampal region were abnormal. These results suggested that cognitive function decline in MCAO mice is accompanied by increased inflammasome expression. In contrast, the aforementioned indices were effectively ameliorated in EP+MCAO group, which indicated that exercise preconditioning could improve the inflammatory response and cognitive impairment in ischemic stroke.

A cross-sectional study indicated that exercise preconditioning was associated with intact cognition in patients [35]. Numerous studies in animals have shown that exercise training can change the composition and functional capacity of the gut microbiota [36–40]. Similarly, population studies found that alpha diversity and relative abundance of 40 different bacterial taxa in the gut microbiota of professional athletes were significantly greater than that of sedentary controls [41]. Notably, there are few studies on the relationship between gut microbiota and PSCI. Until 2020, it was first reported that patients with PSCI had altered microbiota composition and corresponding metabolites and correlated with the degree of cognitive impairment, which suggested that gut microbiota may work essentially in the development of PSCI [42]. Therefore, we also analyzed the composition of the gut microbiota in each group. Our data showed that mice treated with MCAO had an increase in gut microbiota alpha diversity compared to the Sham group. This finding was supported by a prospective cohort study [43], showing that the gut microbiota of poststroke patients has higher alpha diversity than healthy controls. Conversely, we observed a decrease in microbial diversity in MCAO mice pretreated with 4 weeks of voluntary exercise.

We further observed that the *Helicobacter* was decreased and the *Roseburia* was increased in the Sham group compared with the MCAO group. *Helicobacter* is a member of the *Proteobacteria* phylum, which has a proinflammatory effect and is closely associated with ischemic stroke [44]. *Helicobacter* is an endotoxin-producing bacteria that can increase endotoxin and improve intestinal permeability. These changes disrupted the intestinal epithelial barrier, allowing harmful substances to enter the peripheral blood. However, exercise preconditioning significantly enriched beneficial bacteria such as the *Lactobacillus* and *Alistipes* and reduced the *Ruminococcus*. Among these beneficial bacteria, *Lactobacillus* is widely recognized for the role in preserving human health and modifying immune function [45–47]. In addition, *Lactobacillus* has been reported to be protective in a rat model of ischemic stroke by inhibiting neuronal apoptosis, reducing brain infarct volume, decreasing oxidative stress, and restoring neurobehavioral deficits [48]. *Alistipes* is a relatively new genus of bacteria that can be seen as a potential SCFA (short-chain fatty acid) producer [49]. By analyzing SCFAs in the intestinal contents of rats with PSCI, researchers found that concentrations of acetic and propionic acids were lower in the early stroke than in the Sham group, and butyric and valeric acids were consistently at low level [50]. These results suggest that gut microbiota SCFA concentration is associated with the occurrence and prognosis of cognitive impairment after stroke. The mechanism behind the treatment of cerebral ischemic stroke with SCFAs may involve reducing inflammation, remodeling of the gut microbiota [44]. It has been found that intestinal SCFA levels decreased after ischemic stroke. Transplantation of SCFA-rich fecal bacteria and inhibition of inflammation are effective treatments for ischemic stroke [51]. Once the inflammatory response can be inhibited, the progression of neurons death can be alleviated, and cognitive function can be improved after stroke. In this experiment, we found that exercise preconditioning improved poststroke cognitive impairment. We also observed that exercise preconditioning can improve cognitive dysfunction by inhibiting NLRP3 inflammasome as well as enriched *Lactobacillus* and *Alistipes* and reduced *Ruminococcus*. These findings indicated that exercise preconditioning can significantly affect the composition of the gut microbiota by adding beneficial bacteria and reducing hazardous bacteria, thereby inhibiting the activation of inflammasome and attenuating the inflammatory response in mice.

We acknowledge several important limitations to our study; firstly, there is no in-depth study of FMT (fecal microbiota transplantation) on the basis of gut microbiota. FMT has become a research hotspot in the field of basic medicine and clinical medicine and may involve several mechanisms worthy of further exploration: (1) inhibiting the expression of inflammatory factors in intestinal and brain tissues after stroke; (2) increasing the number of beneficial bacteria and restoring the normal structure of intestinal flora; and (3) promoting the expression of intestinal tight junction proteins and reducing intestinal mucosal permeability. Another limitation is that we ignored the resilience of penumbra neurons in our selection of specimens, as the number of collateral vessels that can still supply oxygen and glucose to the neurons of penumbra to prevent irreversible necrosis around necrotic core. Finally, our inability to show certain associations may be due to insufficient sample size, rather than the absence of a true relationship.

## 5. Conclusion

In conclusion, our study suggests that exercise preconditioning can improve cognitive dysfunction after ischemic stroke by alleviating inflammation and regulating the composition and diversity of gut microbiota. The molecular mechanism may involve the inhibition of NLRP3 inflammasome-mediated inflammatory response. This evidence suggests that regulating the composition and diversity of gut microbiota and inhibiting inflammatory response through exercise preconditioning can be an efficient preventive measure in ischemic stroke.

## Data Availability

The datasets used and analyzed during the current study are available from the corresponding author upon reasonable request.
